# Deciphering the role of DOCK8 in tumorigenesis by regulating immunity and the application of nanotechnology in DOCK8 deficiency therapy

**DOI:** 10.3389/fphar.2022.1065029

**Published:** 2022-11-01

**Authors:** Longhui Zhang, Yang Cao, Xiangpeng Dai, Xiaoling Zhang

**Affiliations:** ^1^ Key Laboratory of Organ Regeneration and Transplantation of Ministry of Education, First Hospital of Jilin University, Changchun, China; ^2^ National-Local Joint Engineering Laboratory of Animal Models for Human Disease, First Hospital of Jilin University, Changchun, China; ^3^ Clinical Laboratory, The Eastern Division of the First Hospital, Jilin University, Changchun, China

**Keywords:** dedicator of cytokinesis 8, immunity, tumor, Cdc42, cytoskeleton, NK cells, hematopoietic stem cell transplantation, nanotechnology

## Abstract

The dedicator of cytokinesis 8 (DOCK8) immunodeficiency syndrome is a severe immune disorder and characterized by serum IgE levels elevation, fungal and viral infections, dermatitis and food allergies. It was well known that DOCK8 is crucial for the survival and function of multiple immune related cells. However, the critical role of DOCK8 on tumorigenesis through regulating immunity is poorly investigated. Accumulating evidences indicated that DOCK8 could affect tumorigenesis by regulating the immunity through immune cells, including NK cells, T cells, B cells and dendritic cells. Here, we summarized and discussed the critical role of DOCK8 in cytoskeleton reconstruction, CD4^+^ T cell differentiation, immune synaptic formation, tumor immune infiltration, tumor immune surveillance and tumorigenesis. Furthermore, the potential roles of nanotechnology in improving the hematopoietic stem cell transplantation-based therapy for DOCK8 deficiency diseases are also highlighted and discussed.

## Introduction

The dedicator of cytokinesis (DOCK) protein family are guanine nucleotide exchange factors (GEF) and mainly regulate the activity of GTP enzymes of Rho family, including RhoA, Rac1 and CDC42 (Gadea and Blangy, 2014). Eleven DOCK proteins have been found in mammals and they are divided into four subclasses according to their sequence similarity and functional domain structure: DOCK-A (DOCK1, 2 and 3), DOCK-B (DOCK4 and 5), DOCK-C (DOCK6, 7 and 8) and DOCK-D (DOCK9, 10 and 11) (Nishikimi et al., 2013). Members of the DOCK protein family share two homologous domains, DHR1 and DHR2. The DHR1 domain, located at the N-terminal, can interact with phospholipids and promote the localization of enzyme complexes in the plasma membrane (Yang et al., 2009). Therefore, the DHR1 domain is necessary for downstream signal transduction and biological function. The DHR2 domain, located at the C-terminal, acts as a molecular switch and can catalyze the transformation of Rho family GTP enzymes from the inactivation state binding with GDP to the activation state binding with GTP (Kulkarni et al., 2011).

DOCK8, a well-studied member of the DOCK protein family, is an atypical GEF (Cote and Vuori, 2002). DOCK8 is mainly expressed in the immune system, kidney, lung, placenta, pancreas, and microglia of the central nervous system (Su, 2010; Namekata et al., 2019). DOCK8 immunodeficiency syndrome (DIDS) caused by DOCK8 deficiency is autosomal recessive inheritance. The main clinical manifestations are elevated serum IgE levels, viral and fungal infections, food allergy, dermatitis, eosinophilia, decreased T and B cells, and increased incidence of malignant tumors (Zhang et al., 2009). Mechanismly, DOCK8 regulates the immune response and affects the function of immune cells by regulating the signal transduction of immune cells *via* Rho GTPases. Previous studies have also found that DOCK8 is associated with lymphocyte migration, survival and immune synaptic formation (Randall et al., 2011; Kearney et al., 2017a). In particular, accumulating evidences showed the high correlation between DOCK8 mutations or deletions and cancer development (Takahashi et al., 2006; Kang et al., 2010; Kuskonmaz et al., 2017; Buchbinder et al., 2019). However, the underlying mechanisms of DOCK8 in tumorigenesis are not fully investigated and discussed.

DOCK8 immunodeficiency syndrome (DIDS) is a severe genetic disorder and affected the immune cell migration, survival and functions. However, allogenic hematopoietic stem cell transplantation (HSCT) is the current therapeutic strategy to efficiently treat DOCK8 deficiency related diseases. But the wide application of HSCT is limited by the need of compatible donors, the adequate number of cells, unpredictable adverse effects, immune rejection, graft-versus-host disease (GVHD) and high expenses (Ravendran et al., 2022). Fortunately, nanotechnology was applied in many fields including the pharmaceutical and medical research such as diagnosis (nanodiagnosis), controlled drug delivery (nanotherapy), and regenerative medicine (Soares et al., 2018). Therefore, nano-scaffold, nano-crystals and nano-sized drug delivery device made by nanomaterials might be used to expand the stem cells, increase solubility and improve bioavailability and deliver drugs, respectively (Shen and Wu, 2007; Grattoni et al., 2012; Soares et al., 2018). Hence, the combinations of stem cell therapy and nanotechnology could improve the stem cell based therapy.

Here, we reviewed the common role of DOCK8 in immunity and tumorigenesis, which will help to understand the molecular mechanism of DOCK8 deficiency in cancer development and provide new option for the development of novel therapeutic strategy based on the genetic status of DOCK8.

### DOCK8 regulates cytoskeleton through CDC42 to affect T cell function

There are 20 members of the Rho GTPases superfamily in human and they are divided into five subfamilies according to their structures and functions, including Rho subfamily (RhoA, RhoB and RhoC), Rac subfamily (Rac1, Rac2, Rac3 and RhoG), CDC42 subfamily (CDC42, TC10, TCL, Wrch1 and chp/Wrch2), Rnd subfamily (Rnd1, Rnd3/RhoE and Rnd2) and Rho BTB subfamily (Rho BTB1 and Rho BTB2) (Fukata and Kaibuchi, 2001; Heasman and Ridley, 2008; Hodge and Ridley, 2016). Among them, CDC42, Rac1 and RhoA are the most well-studied Rho GTPases (Schuldt, 2011; Park et al., 2015; Escamilla et al., 2017). In general, Rho GTPases are active when bound to GTP and inactive when bound to GDP. Rho GTPases switch back and forth between these two states and interact with downstream effectors to transmit signals (Kunimura et al., 2020).

**FIGURE 1:**
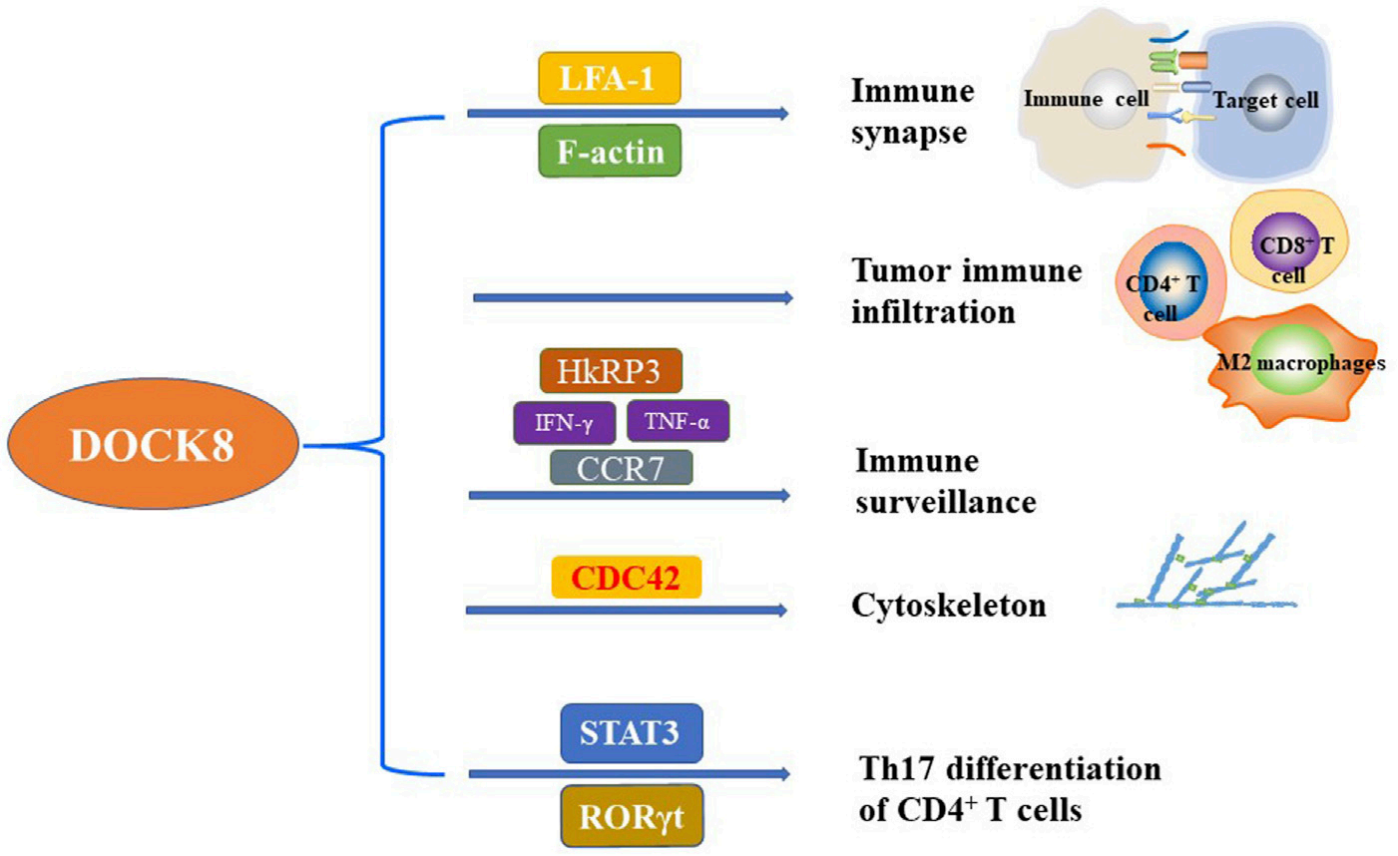
The critical function of DOCK8 on the regulation of immunity. DOCK8 plays important role in regulating the immunity and maintaining the basic function of multiple immune cells. The main function of DOCK8 related to immunity included but not limited to the regulation of immune synapse, tumor immune infiltration, immune surveillance, cytoskeleton, and Th17 differentiation of CD4^+^ T cells.

Wiskott-Aldrich syndrome protein (WASP) is a downstream effector of the Rho GTPase signaling pathway and initiates the assembly of microfilaments by activating the actin related protein 2/3 (Arp2/3) complex, which is a key factor in regulating actin skeleton remodeling (Thrasher and Burns, 2010). It was reported that cytoskeleton plays an important role in cell migration, phagocytosis and immune synapse formation in immune cells and hematopoietic cells (Ham et al., 2013). As a specific GEF of CDC42, DOCK8 can specifically bind to CDC42 and activate Rho GTPase signaling pathway. The activated CDC42 then stimulates actin polymerization and regulates cytoskeleton by activating the Arp2/3 complex through the downstream effector WASP. Studies have shown that WASP is a key regulator of actin cytoskeleton-dependent T cell function in T cell receptor-driven actin polymerization (Blundell et al., 2010). WASP-interacting protein (WIP) can form a signal complex with DOCK8 and WASP, which connects TCR to actin cytoskeleton in TCR-driven actin assembly and promotes T cell migration to secondary lymphoid organs (Janssen et al., 2016). CDC42 can also bind to the myotonic dystrophy kinase-related CDC42-binding kinase (MRCK) (Leung et al., 1998). MRCK plays a role in actin kinetics by stimulating the phosphorylation of myosin-II regulatory light chain (MLC2) (Gomes et al., 2005; Wilkinson et al., 2005). Therefore, DOCK8 might play important role in actin aggregation and cytoskeleton reconstruction by regulating WASP and MRCK through CDC42 (Figure 1).

### Dysregulation of DOCK8 contributes to defective CD4^+^ T cells differentiation

It is well known that the native CD4^+^ T cells can differentiate into five different subtypes, including type 1 Th cells (Th1), type 2 Th cells (Th2), type 17 Th cells (Th17), T follicular helper cells (Tfh) and regulatory T cells (Treg) (Mosmann et al., 1986; Zhu, 2018). Th1 cells mainly target intracellular microorganisms such as bacteria and viruses, but are also involved in some autoimmune diseases (Szabo et al., 2003; Zhu and Paul, 2008; Zhu et al., 2010). Th2 cells mainly target large extracellular microorganisms such as worms, but they are also involved in allergic diseases such as atopic dermatitis, food allergy, allergic rhinitis and asthma (Lambrecht and Hammad, 2009; Paul and Zhu, 2010; Licona-Limon et al., 2013; Zhu, 2015; Walker and McKenzie, 2018). Th17 cells can resist extracellular bacterial infection, but they also play an important role in autoimmune diseases and inflammation (Singh et al., 2014).

An early analysis of patients with autosomal recessive hyper IgE syndrome (AR-HIES) showed that these patients had defects in Th17 differentiation, and some of them were DOCK8 deficiency (McDonald, 2012). The mutations of Signal Transducer and Activator of Transcription 3 (STAT3) in peripheral T cells of autosomal dominant (AD) form of HIES (AD-HIES) patients reduced the expression of RORγt which is essential for Th17 differentiation (Al Khatib et al., 2009; McDonald, 2012). Keles *et al.* have shown that patients with DOCK8 deficiency are defective in Th17 differentiation due to the attenuated interaction of DOCK8 with STAT3 (Keles et al., 2016). Moreover, subsequent studies found that DOCK8-deficient memory CD4^+^ T cells produced less Th1 cytokines (IFN-γ) and Th17 cytokines (IL-17A, IL-17F and IL-22), and more Th2 cytokines (IL-4, IL-5 and IL-13) (Tangye et al., 2017) which implies that memory CD4^+^ T cells are polarized to Th2 phenotype when DOCK8 is defective. However, the underlying mechanisms of type 2 deviation caused by DOCK8 defect is not clear which warrants further in deep investigation (Figure 1).

### DOCK8 is essential for the formation of immune synapses

Immune synapses are transient structures formed between antigen-presenting cells or target cells and lymphocytes (Dustin, 2014; Kearney et al., 2015). The immune synapse formation will promote the activation of immune cells (Dustin, 2002). DOCK8 could regulate synaptic formation by promoting B cell lymphocyte function associated antigen-1 (LFA-1) polarization (Arana et al., 2008; Zhang and Wang, 2012). In the absence of DOCK8, the polarization of LFA-1 is impaired, leading to abnormal synapse formation, making it difficult to maintain B cell germinal centers and impairing the production of long-lived antibodies (Randall et al., 2010). Further study found that DOCK8 promoted the accumulation of adhesion molecules and cytotoxic particles in immune synapses and enhanced the activation of B cell receptor (BCR) (Sun et al., 2019). Furthermore, DOCK8 deficient human NK cells are impaired in the polarization of cytotoxic synapses due to the integration of LFA-1 and F-actin, which subsequently affecting the delivery of cytotoxic particles to target cells and decreasing the cytotoxic activity (Ham et al., 2013; Mizesko et al., 2013). It was also reported that the formation of T cell immune synapse depends on TCR/CD3-driven F-actin recombination (Huang and Burkhardt, 2007) and DOCK8 could connect TCR to actin cytoskeleton in TCR-driven actin assembly through WASP signal whose activation is necessary to the initiation of immune synapses (Janssen et al., 2016). These results suggested that DOCK8 might also indirectly regulate the formation of immune synapses by connecting TCR to actin cytoskeleton and it can be supposed that DOCK8 enhances immune tolerance by promoting the formation of immune synapses in Tregs (Janssen et al., 2017).

In summary, DOCK8 plays an important role in the formation of immune synapses in various immune cells to enhance the killing effect of NK cells and T cells on tumor cells (Figure 1).

### DOCK8 is positively correlated with tumor immune infiltration

Tumor microenvironment (TME) is indispensable for the occurrence and development of tumor. TME includes various cellular components, such as fibroblasts, vascular endothelial cells, adipocytes, immune cells, and non-cellular components, including extracellular matrix and soluble molecules, such as growth factors, cytokines, chemokines and extracellular vesicles (Xiao and Yu, 2021). The infiltration level of immune cells in TME is correlated with the prognosis of tumor. Importantly, in human papillomavirus (HPV) positive head and neck squamous cell carcinoma (HNSCC), the expression of DOCK8 was positively correlated with the level of immune cell infiltration (Zhang et al., 2021). In detail, the expression of DOCK8 had a weak correlation with M1 macrophage markers such as NOS2, IRF5 and PTGS2, but had a strong correlation with M2 macrophage markers such as CD163, VSIG4 and MS4A4A (Kearney et al., 2017a). These results partially explain why HNSCC patients with high DOCK8 expression have a better prognosis. In addition, the clear cell renal cell carcinoma (ccRCC) patients with high DOCK8 expression had better survival time (Wang et al., 2021). It was found that DOCK8 deficient mice showed a lack of infiltrating CD4^+^ T cells in the skin infected with herpes simplex virus (HSV) (Flesch et al., 2015). These results suggest that DOCK8 may be a candidate biomarker for prognosis and a new target for immune-related therapy due to its critical function in tumor immune infiltration (Figure 1).

### DOCK8 is related to tumor immune surveillance by regulating NK and T cells

Immune surveillance, one of the most basic functions of the immune system, is the process that the immune system could recognize, kill and clear mutant cells in the body (Burnet, 1970). NK cells, as part of the innate immune defense against infections and tumors, play a key role in human immune surveillance (Orange, 2013). Some studies indicated that the patients with DOCK8 deficiency showed impaired function of NK cells which cannot be recovered by IL-2 stimulation, due to the inability to form mature immune synapses through F-actin accumulation in targeted synapses (Mizesko et al., 2013). In addition, the Hook-related protein 3 (HkRP3), a novel DOCK8 interacting protein, has been shown to partially mediate the cytotoxicity of NK cells by regulating the aggregation of dissolved particles and the polarization of MT organizing center (MTOC) (Ham et al., 2015). Importantly, DOCK8 promotes the transcription and secretion of IFN-γ and TNF-α in NK cells by promoting the activation of Src kinase Lck (Kearney et al., 2017b). Consistently, lack of DOCK8 not only leads to deficiency in the secretion of IFN-γ in human NK cells, but also impairs the expression of C-C Motif Chemokine Receptor 7 (CCR7) on the surface of NK cells in a WASP-independent manner (Patrizi et al., 2022). Furthermore, DOCK8 deficient NK and T cells cannot coordinate the cytoskeleton structure through CDC42 and p21-activated kinase (PAK), which eventually leads to cytothripsis (Zhang et al., 2014).

CD4^+^ T cells and CD8^+^ T cells play an important role in killing tumor cells (Hunder et al., 2008; van der Leun et al., 2020). CD8^+^ T cells are the main effector cells to kill tumor cells, while activated CD4^+^ T cells can significantly increase the cellular components of innate immunity, such as macrophages and NK cells, by increasing the secretion of IFN-γ (Veatch et al., 2022). DOCK8 is essential for T cell survival. In DOCK8 deficient mice, the number of peripheral T cells were decreased, and the CD4^+^ and CD8^+^ T cells (including memory CD8^+^ T cells) in peripheral blood showed an increased turnover and decreased survival rate (Lambe et al., 2011). Similarly, Randall *et al.* reported that DOCK8 plays an autonomous role in regulating CD8^+^ T cells, and DOCK8 deletion impairs the survival and function of CD8^+^ T cells in humans and mice (Randall et al., 2011). Moreover, DOCK8 deficiency in mice led to the loss of CD44^+^ NK1.1^+^ NKT cells in the thymus, while in the liver, DOCK8 deficient NKT cells expressed lower levels of survival factor B cell lymphoma 2 and integrin lymphocyte function associated antigen 1 (Crawford et al., 2013). Furthermore, although DOCK8 is not necessary for NKT cell activation, it is necessary for CD44^+^ NK1.1^+^ NKT cell proliferation and differentiation (Crawford et al., 2013).

Altogether, DOCK8 is important for the survival and function of NKT cells and T cells which might be the underlying mechanisms for the ability of DOCK8 in regulating immune surveillance (Figure 1).

### The critical role of DOCK8 in various cancers

**FIGURE 2:**
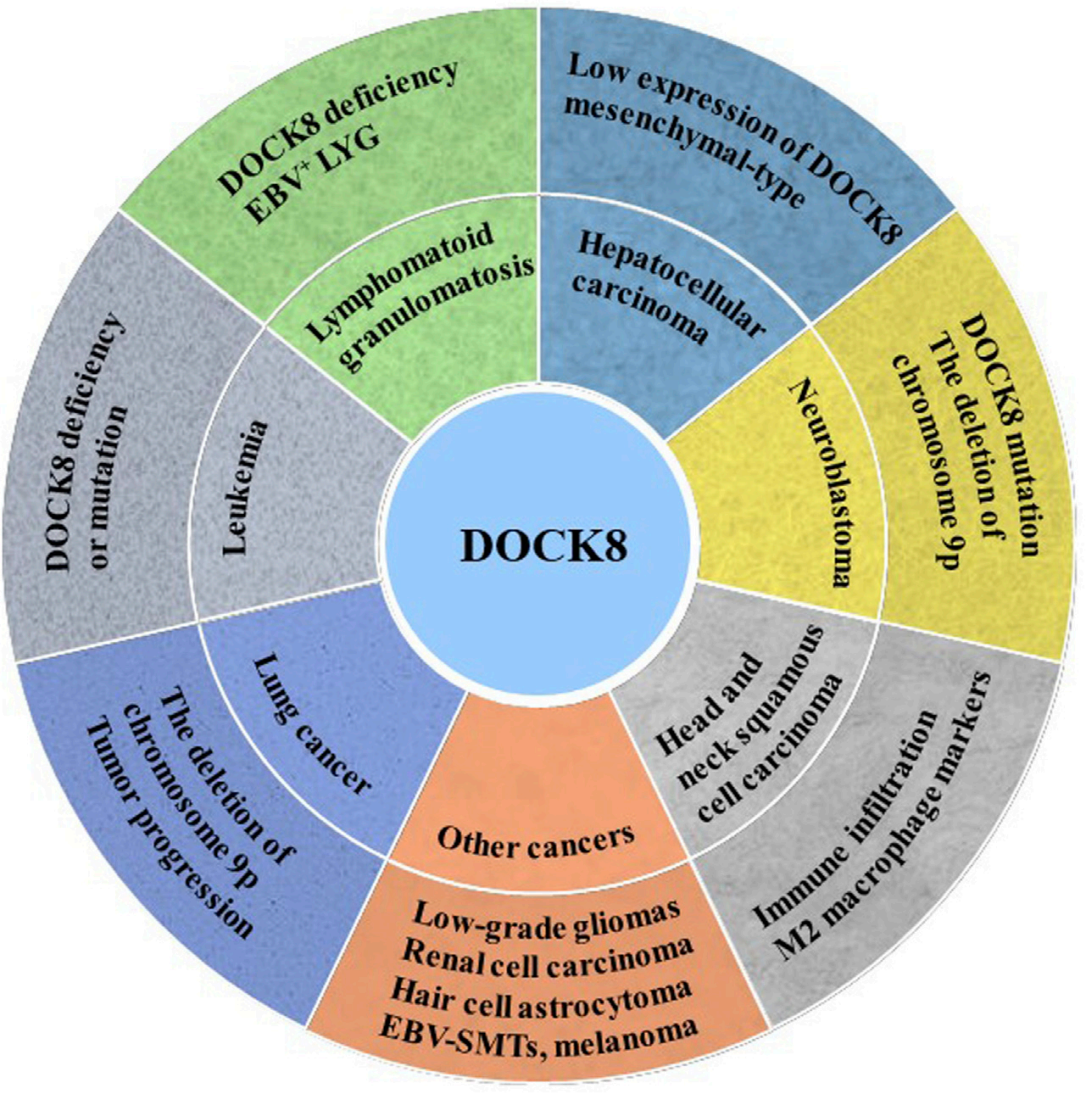
DOCK8 deficiency was associated with tumorigenesis. DOCK8 deficiency might be an important inducer for multiple cancers initiation such as lung cancer, leukemia, lymphomatoid granulomatosis (LYG), hepatocellular carcinoma (HCC), neuroblastoma (NB) and others.

Given that DOCK8 is important for the function of NK, T cell and tumor immune surveillance, DOCK8 might play critical roles in tumorigenesis. Immunodeficiency syndrome, caused by mutations or deletions of the DOCK8 gene, is characterized by persistent microbial infection and increased incidence of malignant tumors. Here, we summarized and discussed the crucial roles of DOCK8 in various cancers (Figure 2).

Leukemia, a malignant tumor of the hematopoietic system, is characterized by excessive production of leukemia cells in the bone marrow and invades most organs of the human body. DOCK8 mutation and deficiency were detected in the B-cell lymphocytic leukemia patient (Buchbinder et al., 2019) and acute myeloid leukemia (AML) patient, respectively (Kuskonmaz et al., 2017) which implies that DOCK8 deficiency or mutation may contribute to the occurrence of leukemia. However, it was also reported that AML patients with high expression of DOCK5/8 have a low survival rate (Biswas et al., 2019) due to the elevated activity of Rac GTPase signal downstream of DOCK5/8.

Lymphomatoid granulomatosis (LYG) is a kind of B lymphocyte proliferative disease closely related to Epstein-Barr virus (EBV). The characteristics of LYG vary greatly between patients. A study reported that a brother and sister are DOCK8 deficiency and the younger sister presented as EBV^+^LYG (Dimitriades et al., 2017). However, the younger sister has only a small number of atopic dermatitis and viral skin infections, while the elder brother shows an increase in typical atopic dermatitis and viral skin infections (Dimitriades et al., 2017). The phenotypic variations in atopic dermatitis and viral skin infections between the siblings may be related to somatic reversal mutations of DOCK8 (Jing et al., 2014).

Hepatocellular carcinoma (HCC) is the main histological subtype of liver cancer, accounting for 90% of primary liver cancer. One study showed that 44% of patients had low expression of DOCK8 gene, and chromosome 10q25.3 allele deletion was an independent prognostic factor for poor survival of HCC patients (Saelee et al., 2009). Wang *et al.* reported that the activated Src tyrosine kinase up-regulates the expression of DOCK8, which promotes the activation of Rac1 (a key molecule of mesenchymal-type movement) and participates in the invasion and metastasis of HCC cells (Wang et al., 2015). Therefore, the low expression of DOCK8 may be related to the occurrence and development of HCC, and the increased expression of DOCK8 can promote the mesenchymal-type movement of HCC cells.

Neuroblastoma (NB) is the most common extracranial tumor in children. Previous studies have found that the amplification, mutation and deletion of key genes are main causes of neuroblastoma occurrence (Brodeur et al., 1984; Cohn et al., 2009; Schulte et al., 2011). An exon sequence result of 16 patients with primary and recurrent neuroblastoma showed that DOCK8 mutations were detected in two patients and deletion of chromosome 9p was detected in another five patients (Schramm et al., 2015). Abnormalities on chromosome 9 of DOCK8 were also found in some high-risk neuroblastoma patients, *in situ* mouse models and 3D neuroblastoma models (Lopez-Carrasco et al., 2020). In addition, it was found that DOCK8 showed a higher expression in mouse stromal Schwann cells (SW10) and lower expression in neuroblastoma cell SK-N-BE 2) (Monferrer et al., 2020).

Lung cancer is one of the most threatening malignancies to human. Lung cancer is divided into non-small cell lung cancer and small cell lung cancer. The non-small cell lung cancer is further divided into three subtypes: squamous cell carcinoma, adenocarcinoma, and large cell lung cancer (Herbst et al., 2008). It is well known that the human DOCK8 gene is on chromosome 9. Importantly, recurrent copy number loss on chromosome 9p in lung squamous cell carcinoma was associated with tumor progression and was preferentially identified in advanced tumors (Kang et al., 2010). Takahashi *et al.* identified chromosome 9p24 pure deletion in a lung cancer cell line and decreased DOCK8 expression which might be caused by DNA methylation or histone deacetylation (Takahashi et al., 2006). Moreover, DOCK8 pure deletion, DMRT1 and DMRT3 deletions on chromosome 9p24.3 were also found in lung squamous cell carcinoma (Kang et al., 2010).

The result from a follow-up study of 136 patients with DOCK8 deficiency showed that 23 patients (17%) were diagnosed with malignant tumors, including 11 cases of hematological cancer, 9 cases of epithelial cancer and 5 cases of other malignant tumors (Aydin et al., 2015). Another study reported that 7 (16.3%) of 43 DOCK8 deficiency patients developed malignant tumors, and the incidence was higher than that of other immunodeficient diseases such as ataxia-telangiectasia and Wiskott-Aldrich syndrome (Cekic et al., 2020). Moreover, DOCK8 was found to be a new candidate gene negatively associated with the progression of low-grade gliomas (Idbaih et al., 2008). In addition, the expression of DOCK8 was significantly decreased in patients with renal cell carcinoma which showed a poor overall survival time than that with high expression of DOCK8 (Shu et al., 2022). Consistently, the expression of DOCK8 is down-regulated in children with hair cell astrocytoma, which may be caused by abnormal methylation (Zhao et al., 2014). The result of a whole-exome sequencing of patients with Epstein Barr Virus smooth muscle tumors (EBV-SMTs) identified a compound heterozygous variant of CARMIL2 and DOCK8 (Yonkof et al., 2020). A combination of meta-analysis of nevus GWAS and melanoma GWAS confirmed that the single-nucleotide Polymorphism (SNPs) of DOCK8 reached a global significant level, although DOCK8 has not been considered an important gene in the pathogenesis of melanoma (Duffy et al., 2018). Interestingly, due to the positive correlation between DOCK8 expression and the level of immune cell infiltration in TME, DOCK8 could be considered a prognostic biomarker for HNSCC (Zhang et al., 2021).

### Potential role of nanotechnology on the improvement of hematopoietic stem cell transplantation based therapy for DOCK8 deficiency diseases

DOCK8 immunodeficiency syndrome (DIDS) is caused by DOCK8 defects such as mutation or deletion. DIDS is a severe genetic disorder with characteristics of increased susceptibility to viral and bacterial infections, atopic diseases and other malignancy such as immune related diseases and tumor. DOCK8 deficiency affected the immune cell migration, survival and functions partially by impairing the actin polymerization and cytoskeletal rearrangement. Therefore, allogenic hematopoietic stem cell transplantation (HSCT) is developed to treat DOCK8 deficiency related diseases. A follow-up survey of 81 patients who received HSCT therapy showed that 68 patients (84%) eventually survived, indicating that HSCT is effective for most DIDS patients (Aydin et al., 2019). And further studies have confirmed that HSCT can effectively restore the differentiation and function of lymphocytes in DIDS patients, corresponding to the improvement of clinical symptoms (Pillay et al., 2019). However, other factors such as the need of compatible donors, the adequate number of cells, unpredictable adverse effects, immune rejection, graft-versus-host disease and high expenses limited the wide therapeutic application of HSCT (Ravendran et al., 2022).

In recent years, nanotechnology has been applied in increasing number of fields including the pharmaceutical and medical research such as diagnosis (nanodiagnosis), controlled drug delivery (nanotherapy), and regenerative medicine (Soares et al., 2018). It is well-known that the small size effects, surface effects, quantum size effects, and tunnel effects are the four basic unique properties of nanomaterials which provide novel technological opportunities especially in combination with other technologies (Wang et al., 2009). Due to the unique properties of nanomaterials, great progress was made for their application in stem cell research including the isolating and sorting of stem cells, molecular imaging and tracing of stem cell transplantation, gene or drugs delivery into stem cells. In particular, the unique nanostructures designed by nanomaterials could be used for the improvement of proliferation and differentiation of stem cells partially by mimicking the cellular environment to affect stem cell behaviors (Soares et al., 2018).

**FIGURE 3:**
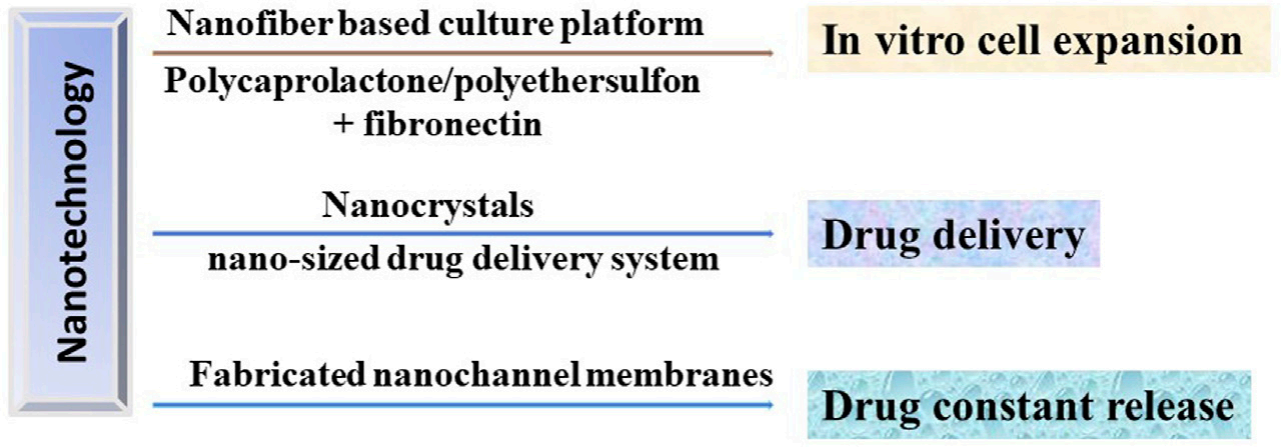
Potential applications of nanotechnology in HSCT in treating DOCK8 deficiency diseases. Nanotechnology could be used to produce the *in vitro* culturing platforms to expand the HSC with high efficiency, the drug delivery and release system to precisely deliver the immunosuppressants to desired therapeutic area and to achieve constant release of drugs, respectively.

In order to obtain sufficient number of hematopoietic stem cells for clinical usage, Mousavi *et al.* designed a three-dimensional polycaprolactone nano-scaffold coated with fibronectin to improve the expansion efficiency and self-renewal ability of umbilical cord blood hematopoietic stem cells *in vitro* (Mousavi et al., 2018). Moreover, CD133+ hematopoietic stem cells could expand on the fibronectin conjugated polyethersulfon scaffold (Eskandari et al., 2015). Furthermore, the nanofiber-based serum-free *ex vivo* expansion culture platform allows the progenitor cell to expanded about 225-fold without differentiation (Das et al., 2009). To reduce the possibility of immune rejection and GVHD occurring during HSCT, immunosuppressive drugs were administrated and significantly improved the transplant patient survival. However, the poor bioavailability and water solubility affected the therapeutic effect of immunosuppressants (Tasciotti et al., 2016). Notably, nanocrystals can be used as a delivery platform to provide increased surface area which leads to increased solubility and improved bioavailability (Shen and Wu, 2007). Furthermore, nano-sized drug delivery technologies could help to deliver drugs into a desired therapeutic area which reducing the necessary drug dosage, adverse effects and toxicity typically associated with conventional delivery system (Grattoni et al., 2012). Additionally, the nanochannel drug delivery system constructed by precision-fabricated nanochannel membranes could achieve constant release of drugs in longer timeframes by briefly changing the channel size (2–200 nm) and density (Grattoni et al., 2012). Therefore, the combinations of stem cell therapy and nanotechnology will achieved great progress in cell engineering and treatment of DOCK8 deficiency diseases (Figure 3).

## Conclusion and outlook

DOCK8 is notorious because of the immunodeficiency syndrome caused by DOCK8 deficiency. The main characteristics of DIDS include elevated serum IgE levels, viral and fungal infections, food allergy, dermatitis, eosinophilia and decreased T and B cells. It then well known that DOCK8 play important role in regulating the biological function of different immune cells. In detail, DOCK8 regulates cytoskeleton reconstruction and affects the subsequent migration of immune cells and the formation of immune synapses (Figure 1). Dysregulation of the DOCK8-CDC42-WASP signaling pathway may impair the reconstruction of the cytoskeleton and lead to immune deficiency.

Importantly, T cells, NK cells and B cells cannot form immune synapses without DOCK8. DOCK8 mediates the cytotoxicity of NK cells and the transcription and secretion of cytokines IFN-γ and TNF-α. Furthermore, high expression of DOCK8 can significantly increase the level of immune cell infiltration in TME of HNSCC and ccRCC patients. However, whether overexpression of DOCK8 in tumors can increase the level of tumor immune infiltration is universal in various cancers warrants further in deep study. Importantly, DOCK8 deficiency affects the survival of immune cells such as T cells and NKT cells in cancer which might be through cytoskeleton reconstruction, CD4^+^ T cell differentiation, immune synaptic formation, tumor immune infiltration and tumor immune surveillance. It was well known that the immune surveillance of the body is an important barrier to prevent tumor occurrence. However, how DOCK8 regulates tumorigenesis is not fully investigated. Generally, mutations or deletions of DOCK8 may provide susceptibility to cancer. Deletion or down-regulation of DOCK8 was detected in leukemia, lung cancer, renal cell carcinoma, low-grade gliomas and childhood hairy cell astrocytoma. Mechanismly, the defect of DOCK8 in immune cells may indirectly lead to tumorigenesis by affecting the immune monitoring function. It has been shown that DOCK8 promotes the mesenchymal-type movement of HCC cells, and the expression of DOCK8 is negatively correlated with the occurrence of HCC. Furthermore, a considerable number of neuroblastoma patients are accompanied with DOCK8 mutation or deletion. As we know, the 3-hydroxyo-aminobenzoic acid (3HAA) was reported to attenuate the anti-tumor immunity by inhibiting Th1 and Th2 cells and increasing regulatory T cells (Platten et al., 2005; Adams et al., 2012). Interestingly, some patients with DOCK8 deficiency showed overexpressed 3HAA and aspartic acid (Jacob et al., 2019). Therefore, DOCK8 might play important role in inhibiting tumorigenesis by regulating immunity through different signal pathways.

Importantly, the allogenic hematopoietic stem cell transplantation (HSCT) is now the only reported therapeutic strategy for the treatment of DOCK8 deficiency related diseases. However, the need of compatible donors, the adequate number of cells, unpredictable adverse effects, immune rejection and GVHD are common problems typically associated with HSCT (Ravendran et al., 2022). Therefore, to achieve better therapeutic effect, many strategies were investigated to solve or reduce these limitations during HSCT. Notably, nanotechnology proved the opportunity to reduce or overcome the adverse effects of HSCT due to the unique characteristics of nanomaterials. For example, nanotechnology is successfully used to achieve the precision localization and constant release of delivered drugs to improve the quality of patient life and to provide a scaffold supporting the expansion of stem cells *in vitro*. Furthermore, the patient derived hematopoietic stem cell could be subjected to genetic editing to correct the DOCK8 mutation and then perform the HSCT. Notably, the nanotechnology could also be used to improve the genetic editing efficacy and accuracy. Mout *et al.* assembled nanoparticles with Cas9 protein and sgRNA as a delivery system to achieve 90% delivery efficiency and 30% gene editing efficiency in tested cell types (Mout et al., 2017). Wang *et al.* developed PEGylated nanoparticles based on cationic α-helical polypeptide to achieve an intracellular gene editing of 47.3% which is superior to the traditional polycation transfection agent (Wang et al., 2018).

Furthermore, because DIDS is a disease caused by a single gene defect, correcting the disease-driving mutation in the cells of DIDS patient by genetic therapies to restore the function of DOCK8 might be an alternative treatment for DOCK8 deficiency (Ravendran et al., 2022). Recently, CRISPR/Cas9 and other related technologies are wided used to achieve genetic edition and make huge progress. Importantly, several CRISPR/Cas-based gene editing strategies have been proposed to be used in DIDS, providing a more accurate, lower-risk treatment option for the patients with DIDS (Ravendran et al., 2022). However, many concerns for the genetic editing technologies warrant further in deep investigation before they are applied in the clinical trials for the treatment of DOCK8 deficient patients. How the nanotechnology promotes the therapeutic effect of cell transplantation and genetic editing should also be fully studied.

In summary, the role of DOCK8 on cytoskeleton reconstruction, CD4^+^ T cell differentiation, immune synaptic formation, tumor immune infiltration and tumor immune surveillance might be the main mechanisms for the function of DOCK8 on tumorigenesis. Therefore, this review is helpful to understand the underlying molecular mechanisms of DOCK8 in regulating immunity and tumorigenesis and proved novel option for the development of new therapeutic strategies based on the DOCK8 genetic status.
